# Study on Key Factors Influencing Coordination Effectiveness of Food Safety Coordinating Body: Lessons from the Chinese Context

**DOI:** 10.3390/foods13020289

**Published:** 2024-01-16

**Authors:** Liangyun Niu, Linhai Wu

**Affiliations:** 1 School of Economics, Anyang Normal University, Anyang 455000, China; nly2000nly@163.com; 2 Institute for Food Safety Risk Management, Jiangnan University, Wuxi 214122, China

**Keywords:** food safety, coordination effectiveness, coordinating body, DANP, China

## Abstract

Many countries have established food safety coordination agencies to strengthen cooperation between government agencies. Due to different national conditions, there are differences in the coordination agencies of different countries, but there are also many similarities. This article studies the key factors influencing the coordination effectiveness of China’s food safety coordinating body, so as to enhance interagency collaboration. The results show that the coordination ability of the coordinating body’s head, the authority degree of the coordinating body, the clarity degree of the agency’s legal responsibility, and the clarity degree of the coordination goal are key factors influencing the coordination effectiveness. The conclusions indicate that the key factors influencing the coordination effectiveness of China’s food safety commissions are similar to the key factors in Western countries, without evident differences due to national situations, social systems, and cultures. This article will be useful to policymakers and public sector managers in terms of understanding which key factors influence the coordination effectiveness of coordinating agencies. Moreover, this study provides a perspective for the academic circle from which to understand the main problems faced in the interagency collaborative governance of food safety risks in China.

## 1. Introduction

Food safety is a major public health concern related to human health. As of 19 May 2022, the WHO website states that contaminated or hazard-containing food can cause over 200 different diseases or ailments, and that, annually, 600 million people—almost 1 in 10 people worldwide—fall ill after eating contaminated food, and 420,000 die [1]. A powerful regulation system is the key to ensuring food safety [2]. However, a single government agency is unable to undertake all the regulation functions. Therefore, many countries use systems of joint regulation by multiple government agencies based on different functions, which, however, causes the problems of the fragmentation, inconsistency, and overlap, among others, of the functions across government agencies to different extents in actual regulation [3]. Many countries have established government coordinating bodies as a device in the forms of commissions, working groups, task groups, and so on, to promote the food safety risk governance effectiveness through interagency collaboration [4,5]. For instance, in the United States, where food safety regulations are effective, approximately 15 government agencies operate at the federal level, each responsible for distinct food safety regulatory functions [6]. However, since 2007, the fragmented federal food safety regulation has been identified as a high-risk area by the U.S. GAO in several reports submitted to Congress. Consequently, the President’s Food Safety Working Group was established in 2009 as a coordinating body for all regulatory agencies responsible for food safety affairs in the federal government [7].

Since the 1980s, for the purpose of improving the regulation effectiveness, the Chinese government has carried out eight rounds of reforms to the food safety regulation system; however, there are still more than 10 agencies undertaking different regulation functions at each of the four levels of government in the framework system, namely, from the central government to the county-level government [7,8]. The fragmentation of regulation by multiple government agencies is also regarded as one of the core causes of the relatively low effectiveness of the Chinese government’s regulation [8,9]. For this reason, the Chinese government established the Food Safety Commission of the State Council, which comprises 15 central government agencies and is led by three vice premiers of the State Council. The commission serves as the central government’s supreme advisory and coordinating body for food safety. Since 2010, all governments at three levels in China (i.e., provincial governments, prefectural governments, and county-level governments) have established their own food safety commissions, forming a system of food safety coordinating bodies with Chinese characteristics. China’s experience has shown that establishing an independent food safety coordination agency can effectively promote cooperation between regulatory authorities, alleviate the fragmentation of regulation, and improve the regulatory effectiveness. This also provides evidence for other countries to establish food safety coordination agencies.

The 20th CPC National Congress set the goal of a Chinese path to modernization, emphasizing the continuous enhancement of food safety regulation, which requires the further exertion of the overall coordination function of the food safety commissions at all government levels. Therefore, it is necessary to study which dimensions and factors influence the coordination effectiveness of China’s food safety commissions. Moreover, these dimensions and factors exhibit interactive and mutual-influence relationships, forming a multi-dimensional and complex system. In this complex system, what are the mutual relationships among the dimensions and factors? What are the key dimensions and factors? Unfortunately, there are few relatively complete literature reports addressing these questions. Taking China’s food safety commissions as an example, this paper identifies four dimensions and 12 factors influencing the coordination effectiveness of the food safety coordinating bodies, and it then delineates the causal relationships among the different dimensions and factors influencing the coordination effectiveness. Then, the key dimensions and factors influencing the coordination effectiveness are analyzed using the DANP method, which combines the decision-making trial and evaluation laboratory (DEMATEL) method with the analytic network process (ANP) method.

## 2. Food Safety Coordinating Body: Literature Review

### 2.1. Establishment Background: An Important Reform Embracing Post-New-Public-Management Campaign

Since the 1980s, Western countries have launched new public management (NPM) campaigns to overcome the problem of inefficiency in public service caused by the welfare system. This has involved establishing specialized government agencies with single functions; however, new problems, including the government agencies’ selfish departmentalism and service fragmentation, have occurred unexpectedly as a result [10]. Consequently, since the mid–late 1990s, Western countries have been carrying out post-new-public-management (post-NPM) campaigns represented by overall government reform in order to address the disadvantages brought about by the NPM campaigns [11,12]. Collaborative governance, which has been advanced as a tool to improve public service delivery, is widely discussed in the mainstream public administration literature [13]. Establishing government coordinating bodies to enhance the totality of public agencies is one important aspect of the post-NPM campaign [14]. A comparative study of the cross-sectoral post-NPM reforms in 13 countries from 1980 to 2014 showed that policy integration and administrative coordination have grown stronger over the last 20 years [15].

China has made five progressive government agency reforms (in 1982, 1988, 1993, 1998, and 2003) covering the whole country by referencing the experience of Western countries in their NPM campaigns. Although the reforms’ achievements were evident, negative effects similar to those of the NPM campaigns in Western countries occurred [16], such as disputes between government agencies and administrative inefficiency. In a bid to address these issues, China carried out three government agency reforms (in 2008, 2013, and 2018) referencing the West’s experience in their post-NPM campaigns. These reforms included merging government agencies with overlapping responsibilities to build superdepartments and commissioning coordinating bodies beyond the interests of single government agencies to coordinate major matters in economic and social affairs management in an overall comprehensive manner [16,17].

Similar to the situation in Western countries, the establishment of food safety commissions at all government levels in China is also an important reform embracing the post-NPM campaign in the field of food safety, with the purpose of strengthening the coordination and collaboration among different government agencies. However, there are some differences between the food safety coordinating bodies in China and those of Western countries. For example, in the U.S., the food safety regulation functions are mainly undertaken by central government agencies, so the U.S. primarily sets a food safety coordinating body at the federal government level. In China, all provincial-, prefectural-, and county-level regulation agencies undertake their own food safety regulation responsibilities in their corresponding jurisdictions according to their functions, so specialized coordinating bodies are needed in provincial-, prefectural-, and county-level governments.

### 2.2. Core Characteristic: Integration of Hierarchical Coordination and Networked Coordination 

A food supply chain starts with agricultural production, passes through links such as processing, storage, and transportation, and then reaches the consumption link; as such, it encompasses numerous production and operation subjects. Past international experience has shown that it is impractical to incorporate the regulation of all production and operation subjects in the food supply chain into one superdepartment of government [18]. Therefore, since 2004, when the State Council of the PRC established a system whereby multiple government agencies jointly regulate food safety, China has maintained a system of joint regulation by multiple government agencies, although the system has been widely criticized and undergone multiple rounds of reform. At present, the agencies for agriculture and rural affairs, commerce, market regulation, and health, among others, are the main regulatory agencies in China. This correspondingly leads to a difficult problem: how to ensure that the different government agencies work together.

The definition of coordination used here is ‘the adjustment of actions and decisions among interdependent actors to achieve specified goals’. Coordination mechanisms are key to ensuring the realization of collaboration among different government agencies [19], and they mainly include two coordination modes, namely, hierarchical coordination and networked coordination [20]. Coordination through a hierarchy involves political and administrative leaders using instrumental authority to ensure control and guide the formulation and achievement of goals [21]. Such a coordination mode typically comes at the cost of ignoring or even damaging the initiatives of subordinate agencies. Networked coordination mainly functions to horizontally coordinate the shared public affairs of government agencies belonging to the same government level based on mutual dependence and trust, and it compensates for the defects of hierarchical coordination to a certain extent. Network arrangements may be necessary to help mediate departmental conflicts or interests that crosscut policy areas and are devised to forge coordination when a hierarchical mode of coordination is less viable [20]. In fact, hierarchical coordination and networked coordination are not mutually exclusive but have relationships of learning from and merging with each other and existing in parallel [14].

In the system composed of food safety commissions at all government levels and incorporating all relevant government agencies, different local government agencies at the same level work together to solve food safety problems based on mutual dependence and trust. Therefore, such food safety commissions have the characteristic of networked coordination and reflect the horizontal coordination among different public organizations [12]. In addition, as the supreme coordinating body of the government at the corresponding level, a food safety commission with a hierarchical characteristic can vertically coordinate the collaboration among subordinate government agencies through the mechanism of giving commands [22], and then the food safety commission also has the characteristic of hierarchical coordination. Consequently, at all levels of government in China, each food safety commission is a hybrid coordinating body integrating hierarchical coordination and networked coordination. Although the national conditions vary, overall, the food safety coordination agencies in other countries are similar to those in China, being hybrid coordinating bodies integrating both hierarchical coordination and networked coordination.

### 2.3. Main Function: Reducing Transaction Costs of Interagency Collaboration

Transaction cost theory provides a unique theoretical perspective for understanding the function of the government’s food safety coordinating body. A transaction cost can refer not only to economic transaction activities in the market but also to the actions of different government agencies [23]. Owing to the division of labor based on specialization, different government agencies own and accumulate certain specialized skills and action resources; however, a single government agency typically fails to own the resources needed for the completion of some tasks, which can therefore only be completed through joint collaboration among multiple government agencies [24]. This requires breaking through the barriers of the functional dissociation and fragmentation of different government agencies so that they are committed to solving common problems [25].

However, interagency collaboration involves transaction costs, which mainly include the cost of continuous negotiation and consultation due to a lack of consensus among different government agencies and decision inefficiency [26], the time cost incurred because of the need for a more authoritative upper-level government agency to solve disputes, and the emotional cost of depression, distrust, and so on, caused by the failure to identify the other party’s goodwill and collaborative responsibilities in the collaboration [27]. Therefore, efforts to improve interagency collaboration have focused on reducing the transaction costs to make collaborative work easier and progress more quickly. This requires establishing effective mechanisms to improve interagency collaboration, thereby reducing the transaction costs of collaboration [28,29]. This is the main purpose for which Western countries establish food safety coordinating bodies. China’s food safety commissions also utilize an organization structure intended to reduce the transaction costs of interagency cross-boundary collaboration, which raises the effectiveness of interagency collaboration to a certain extent [23,30].

## 3. Dimensions and Factors Influencing Coordination Effectiveness of Food Safety Coordinating Body

Interagency coordination refers to coordination among organizations, and the purpose of establishing a coordinating body is to promote and aim to achieve interagency cross-boundary collaboration [19]. Therefore, studies that have examined interagency collaboration and coordination are closely related to this paper. Based on reviewing the literature in the two aforementioned areas and taking the Chinese context into account, this paper identifies the main dimensions and factors influencing the coordination effectiveness of the food safety coordinating body, as presented in Table 1.

### 3.1. Supporting Capability of Legal System Guaranteeing Coordination

#### 3.1.1. Clarity Degree of Agency’s Legal Responsibility

A legal basis is a prerequisite for government agencies to perform their responsibilities [31]. In China, governments primarily define the responsibilities of the different food safety regulation agencies using methods such as binding laws, regulations, and normative documents. Nevertheless, owing to the food supply chain’s complexity, it is typically hard to clearly and accurately delimit the responsibilities of the different food safety regulation agencies. Moreover, unexpected situations often occur in actual regulation, so responsibility overlaps or blanking among agencies are inevitable. Overlapping responsibilities may result in the phenomenon in which ‘if a matter is profitable, all relevant agencies compete for addressing it, and if a matter is unprofitable, all relevant agencies will push it outward to other agencies’, while responsibility blanking may cause a dilemma wherein no agency is willing to or shall address a particular matter [32,33]. Unclear definitions of government agencies’ responsibilities reduce the regulation effectiveness, and solving problems, such as responsibility overlapping and blanking, requires a coordinating body to promote interagency coordination and collaboration [34].

#### 3.1.2. Operability of Laws and Regulations

The legislation capability and quality of legislative institutions influence interagency cross-boundary governance [35]. Laws and regulations should define the requirements for interagency collaboration as specifically as possible and incorporate all government agencies related to food safety regulation into the cross-boundary collaboration framework [16]. However, many laws, regulations, and government normative documents in China only give principles for the coordination functions of the food safety commissions and interagency collaboration and do not offer clear provisions for the working functions, responsibilities and authorities, and coordination methods. This means that they have low operability in practice, resulting in coordination inefficiency.

#### 3.1.3. Degree of Attention to Common Goals

Government regulatory agencies often pay more attention to their own goals for the purposes of their own benefits and ignore the common goals in interagency collaboration [36]. Scanty attention to common goals reduces the coordination effectiveness [37]. In China, food safety commissions at all government levels issue administrative directive documents every year, which specify their specific annual goals as well as those to be achieved through interagency collaboration. However, the former are dominant, and the weight of the interagency common goals in assessment is relatively low. This is a relatively important factor that influences the coordination effectiveness of China’s food safety commissions.

### 3.2. Capability Characteristic of Coordinating Body

#### 3.2.1. Authority Degree of Coordinating Body

An authoritative coordinating body is the key to improving coordination and achieving effective interagency collaboration [19,38]. Insufficient authority makes it difficult for a food safety coordinating body to address the differences between different government agencies and causes it to lack the sufficient ability to hold government agencies with poor collaboration accountable. In China, prior to 2013, food safety commissions at all levels were independent working agencies with independent internal sections and staffing. However, after 2013, there were no longer independent agencies and dedicated workers for food safety coordination. In the 2013–2018 period, the coordination function was undertaken by the newly founded China Food and Drug Administration, which was superseded by the newly founded State Administration for Market Regulation in 2018 [33]. These changes all served to reduce the coordinating body’s authority and coordination effectiveness.

#### 3.2.2. Clarity Degree of Coordination Goal

If there is no definite and clear coordination goal, the coordinating body and collaborative agencies will find it hard to actively and effectively perform collaboration, which can, in turn, cause the phenomenon of ‘organized irresponsibility’ [14]. Consequently, the coordinating body should have a definite and clear goal for guiding different government agencies to design better collaborative schemes and work with each other, thereby raising the level of cross-boundary collaboration [28,29,39].

#### 3.2.3. Coordination Ability of Coordinating Body Head

In general, a food safety coordinating body implements an administrative-head responsibility system, and the same is true for China’s food safety commissions. The professional quality of the administrative head is crucial to performing the coordinating body’s functions. In order to effectively promote coordination, the administrative head should possess a universally recognized authority and a personal, professional ability [22]; good interpersonal resources, communication skills, and administrative leadership; and special characteristics such as ‘implementing’, ‘following up things’, ‘being an engine in the work’, ‘liking improvement work’, ‘being good at initiating things and local improvements’, ‘working systematically’, and ‘being used to talking in front of a group of people’ [19].

### 3.3. Individual Characteristics of Government Agency in Coordination System

#### 3.3.1. Degree of Difference in Positions among Government Agencies

In a system composed of government agencies at the same level, the positions, functions, and so on, are not completely identical among the different government agencies. For example, in the U.S., the USDA has more power than the FDA [40]. In China, although different regulatory agencies can have the same administrative level, their positions and possessed resources will differ. The market regulation agency is the leading agency, with irreplaceable discursive power, whereas agencies such as commerce, agriculture, rural affairs, and so on are in subordinate positions. Because the mastered resources as well as authorities and responsibilities are different among the various agencies in the food safety regulation system, these agencies also differ in terms of their enthusiasm and initiative for acting as a coordinating body [8].

#### 3.3.2. Cognitive Level of Government Agencies Regarding Resource Dependency

No matter which system is implemented, government regulatory agencies should be established according to the principle of specialization and provided with specialized resources and capabilities [41]. According to resource dependency theory, when a government agency possesses insufficient specialized resources and capabilities and believes that other government agencies have more resources that can make up for its insufficiency, that government agency has a stronger willingness to participate in interagency coordination and collaboration [36].

#### 3.3.3. Degree of Government Agency’s Concern with Declining Power

All government agencies are keen to protect their own scopes of power [42]. In NPM reform actions, a government agency will typically avoid infringing upon the authority and jurisdiction scopes of other government agencies as much as possible [43]. However, interagency collaboration causes government agencies to become concerned with the possibility of the weakening of their own power and to focus on protecting their own power scopes, so that their willingness to collaborate decreases substantially [37]. The same is true in China. Weak and small government agencies tend to protect their own powers from being eroded and ensure that they are not marginalized in the system of government agencies. This impacts the coordination effectiveness of the coordinating body.

### 3.4. Cultural Environment Encouraging Coordination

#### 3.4.1. Loss Degree of Trust Culture

A government agency’s organizational culture is the internal impetus for it to participate in cross-boundary collaboration [44]. To a certain extent, a coordination mechanism’s effectiveness depends on whether different agencies reduce traditional bureaucracy and foster an environment that advocates trust. Interagency trust culture can be regarded as a kind of resource that can reduce the opportunistic behaviors caused by the uncertainty of resource exchange, asymmetric information, and so on, during cross-boundary collaboration [11,45], thereby decreasing transaction costs [28].

#### 3.4.2. Loss Degree of Collaborative Environment

A good collaborative environment among government agencies can effectively improve their coordination effectiveness [20]. However, in the NPM campaign, different government agencies advocate for the decentralization of power and form autonomous cultures on the basis of such power decentralization. This type of autonomous culture is sticky; once it is formed, it is typically hard to change rapidly with changes in the system environment and may generate path dependence, thereby having both real and potential adverse influences on the improvement in the coordination effectiveness [8,21].

#### 3.4.3. Concern Degree from Social Sentiment

The purpose of the constant reforms to public management campaigns is to address public dissatisfaction with the government’s governance of complex public social affairs. Food safety will always rank as a public affair of high social concern. Following a series of dangerous food safety incidents, the public confidence in governments’ food safety risk governance capabilities has been seriously eroded [46]. Governments are urgently seeking new and more effective food safety risk governance methods to respond to public expectations as well as pressure from the media and public opinion [47]. In China, the higher the number of food safety incidents, the more citizens will become concerned about food safety, the more frequently tipoffs will be made, and the higher the coordination work intensity of the food safety commissions at all levels will become; thus, the coordination effectiveness will be higher.

## 4. Data Sources and Research Methods

### 4.1. Data Sources

In this study, we designed a questionnaire according to the set of dimensions and factors in Table 1, which requires expert respondents to score the relationships between every two factors according to the following criteria: ‘No Influence’, ‘Very Low Influence’, ‘Low Influence’, ‘High Influence’, and ‘Very High Influence’, which correspond to 0, 1, 2, 3, and 4 points, respectively. These questionnaires provided the raw data needed for the DANP method.

The DANP method does not require a large number of experts to participate in the evaluation. The average deviation rate (i.e., $\frac{1}{n(n-1)}\sum_{i=1}^{n}\sum_{j=1}^{n}\frac{\left|a_{ij}^{p}-a_{ij}^{p-1}\right|}{a_{ij}^{p}}\times 100\%$ ) was used to determine the number of experts participating in the evaluation by referencing the research effort by Huang et al. [48], where p is the number of experts, $a_{ij}^{p}$ is the average influence of factor i on factor j , and n is the number of influence factors set in the study. The research data in this paper were drawn from nine experts familiar with the coordination functions and actual coordination statuses of China’s food safety commissions. They were from provincial food safety committees, such as those of Jiangxi and Guangxi, and well-known universities, such as Jiangnan University, Shanghai Ocean University, and Zhejiang Gongshang University. In this paper, $\frac{1}{n(n-1)}\sum_{i=1}^{n}\sum_{j=1}^{n}\frac{\left|a_{ij}^{p}-a_{ij}^{p-1}\right|}{a_{ij}^{p}}\times 100\%$ = 3.54% < 5%, indicating that the invited nine experts fulfilled the quantity requirement.

### 4.2. Research Methods

Referencing the research efforts of Huang et al. [48] and Tamura and Akazawa [49], the main calculation process was as follows.

#### 4.2.1. Obtain an Influential Network Relationship Map (INRM) That Shows the Relationships among the Different Dimensions and Factors Influencing the Coordination Effectiveness Using the DEMATEL Method

Step 1: Calculate the average direct-relation matrix (*A*). Firstly, a direct-relation matrix was generated based on the assessment results of each expert member. The direct-relation average matrix ( $A={\left[a_{ij}\right]}_{n\times n},i,j=1,2,\ldots \ldots ,n$ ) was then obtained by calculating the average of the same factor of all the direct-relation matrices (calculation results are given in Table 2).

Step 2: Calculate the normalized initial direct-influence matrix (*D*) as follows (calculation results are given in Table 3):

$$D=z\times A;\;z=\min\left\{1/\max_{i}\sum_{j=1}^{n}a_{ij},1/\max_{j}\sum_{i=1}^{n}a_{ij}\right\},$$

where $i,j\in \left\{1,2,\ldots \ldots ,n\right\}$ .

Step 3: Calculate the total-influence matrix (*T*), $T={\left[t_{ij}\right]}_{n\times n},i,j=1,2,\cdots \cdots ,n$ , where $t_{ij}$ denotes the direct- and indirect-influence degrees of factor i on factor j , as follows (calculation results are given in Table 4):

$$T=D+D^{2}+D^{3}+\ldots +D^{h}=D\left(I-D^{h}\right){\left(I-D\right)}^{-1}$$

$$D={\left[d_{ij}\right]}_{n\times n},0\leq d_{ij}<1,0\leq \sum_{i}d_{ij}\leq 1,0\leq \sum_{j}d_{ij}\leq 1,$$

where $h\to \infty ,D^{h}={\left[0\right]}_{n\times n}$ , $T=D{\left(I-D\right)}^{-1}$ .

Step 4: Calculate the sum of each row and each column of the total-influence matrix (*T*) as follows:

$$r_{i}=\sum_{j=1}^{n}t_{ij};\;c_{j}=\sum_{i=1}^{n}t_{ij}$$

where *r_i_* denotes the sum of the direct- and indirect-influence degrees of factor *i* on all of other factors in the system; and *c_j_* denotes the total of the direct and indirect influences that factor *j* receives from the other factors in the system. When *i* = *j*, *r_i_* + *c_i_* represents the total influence that factor *i* exerts and receives from the other factors; *r_i_* − *c_i_* represents the difference in the influence that factor *i* exerts and receives from the other factors; *r_i_* − *c_i_* > 0 indicates that factor *i* has an influence on the other factors and is a causal factor in the system; and *r_i_* − *c_i_ <* 0 indicates that factor *i* is influenced by the other factors and is a result factor in the system. The calculation results are provided in Table 5.

**Figure 1:**
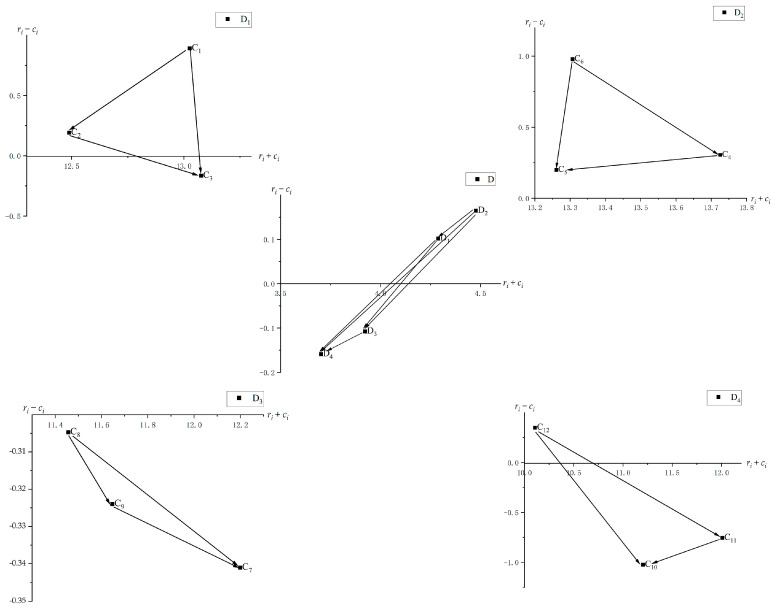
Influential network relationship map of dimensions and factors that influence coordination effectiveness of food safety coordinating body.

Step 5: Obtain an INRM. Based on the (*r_i_* + *c_i_ r_i_* − *c_i_*) of each dimension and factor in Table 5, an INRM of the dimensions and factors can be obtained, as shown in Figure 1.

#### 4.2.2. Obtain Hybrid Weight Using DEMATEL and ANP

Step 1: Obtain an unweighted supermatrix. Firstly, based on the dimensions and factors in Table 1, divide the total-influence matrix (*T*) into matrix $T_{D}$ (dimension-based) and matrix $T_{C}$ (factor-based):

TC= D1⋮c1m1c11 ⋮ Di⋮cimic i1 ⋮ Dn⋮cnmncn1TC11⋯TC1j⋯TC1n⋮ ⋮ ⋮TCi1⋯TCij⋯TCin⋮ ⋮ ⋮TCn1⋯TCnj⋯TCnnD1C11⋯C1m1⋯DiCj1⋯Cjmj⋯DnCn1⋯Cnmn; TD=tD11⋯tD1j⋯tD1n⋮ ⋮ ⋮tDi1⋯tDij⋯tDin⋮ ⋮ ⋮tDn1⋯tDnj⋯tDnn 

Secondly, convert the total-influence matrix ( $T_{C}$ ) into a standardized total-influence matrix ( $T_{C}{\;}^{\alpha }$ ):

TC α= D1⋮c1m1c11 ⋮ Di⋮cimic i1 ⋮ Dn⋮cnmncn1TCα11⋯TCα1j⋯TCα1n⋮ ⋮ ⋮TCαi1⋯TCαij⋯TCαin⋮ ⋮ ⋮TCαn1⋯TCαnj⋯TCαnnD1C11⋯C1m1⋯DiCj1⋯Cjmj⋯DnCn1⋯Cnmn; TC α11=tc11 11/dc1 11⋯tc1j 11/dc1 11⋯tc1m1 11/dc1 11⋮ ⋮ ⋮tci1 11/dci 11⋯tcij 11/dci 11⋯tcim1 11/dci 11⋮ ⋮ ⋮tcm11 11/dc1 11⋯tcm1j 11/dcm1 11⋯tcm1m1 11/dcm1 11,dci 11=∑j=1m1tij 11,i=1,2,⋯m1

Accordingly, obtain an unweighted supermatrix ( W ) of the different factors as follows (calculation results are given in Table 6): $W={\left(T_{C}^{\alpha }\right)}^{\prime }$ .

Step 2: Obtain a weighted standardized supermatrix ( $W^{\alpha }$ ) based on W as follows: (calculation results are given in Table 7):

$$T_{D}{\;}^{\alpha }=\left[\begin{array}{ccccc} t_{D}^{11}/d_{1} & \cdots & t_{D}^{1j}/d_{1} & \cdots & t_{D}^{1n}/d_{1}\\ \vdots & \vdots & \vdots & \vdots & \vdots \\ t_{D}^{i1}/d_{i} & \cdots & t_{D}^{ij}/d_{i} & \cdots & t_{D}^{in}/d_{i}\\ \vdots & \vdots & \vdots & \vdots & \vdots \\ t_{D}^{n1}/d_{n} & \cdots & t_{D}^{nj}/d_{n} & \cdots & t_{D}^{nn}/d_{n} \end{array}\right]=\left[\begin{array}{ccccc} t_{D}^{\alpha 11} & \cdots & t_{D}^{\alpha 1j} & \cdots & t_{D}^{\alpha 1n}\\ \vdots & \vdots & \vdots & \vdots & \vdots \\ t_{D}^{\alpha i1} & \cdots & t_{D}^{\alpha ij} & \cdots & t_{D}^{\alpha in}\\ \vdots & \vdots & \vdots & \vdots & \vdots \\ t_{D}^{\alpha n1} & \cdots & t_{D}^{\alpha nj} & \cdots & t_{D}^{\alpha nn} \end{array}\right],\;d_{ci}^{11}=\sum_{j=1}^{m_{1}}t_{ij}^{11},i=1,2,\ldots m_{1},\;W^{\alpha }=T_{D}^{\alpha }W$$

Step 3: Raise the weighted supermatrix ( $W^{\alpha }$ ) to powers by multiplying it by itself; that is, $\underset{g\to \infty }{\lim}{\left(W^{\alpha }\right)}^{g}$ , until the result converges to a stable-limit supermatrix ( $W^{*}$ ) (calculation results are given in Table 8).

Step 4: Calculate the hybrid weight of each factor with the hybrid-weight formula Z=w+T×w=(I+T)w , and then calculate the normalized hybrid weight of each dimension and each factor, as detailed in Table 9. Here, *z* denotes the hybrid weight, and *w* denotes the comprehensive weight of the secondary indicators in the supermatrix.

## 5. Results and Discussion

### 5.1. Interrelationships between Different Dimensions

Table 5 indicates that the supporting capability of a legal system guaranteeing coordination (D_1_) and the capability characteristic of the coordinating body (D_2_) have positive *r_i_* − *c_i_* values, so they are causal dimensions in the system. This, in turn, indicates that these two dimensions have different degrees of influence on all the other dimensions in the system. The self-characteristic of the government agency in the coordination system (D_3_) and the cultural environment encouraging coordination (D_4_) have negative *r_i_* − *c_i_* values, so they are result/outcome dimensions. Accordingly, these two dimensions are mainly influenced by the other dimensions in the system.

Figure 1 shows that the influence relationships among the four dimensions are as follows: D_2_→D_1_→D_3_→D_4_; D_1_→D_3_→D_4_; D_3_→D_4_ (the arrows denote the influence direction and hold this same meaning below). Dimension D_2_ has the largest *r_i_* − *c_i_* value among all the dimensions. It has the highest influence on the coordination effectiveness and has influences on all the other dimensions. This conclusion also reveals the main root cause of the insufficient interagency coordination and cross-boundary collaborative governance by multiple government agencies in China, and it completely agrees with the country’s actual situation. The natures and functions of China’s food safety commissions at all government levels have undergone almost perpetual change, leaving the food safety commissions’ own capabilities in flux and causing them to fail to meet objective, real demands. Dimension D_4_ is influenced most greatly by the other three dimensions, indicating that improvement in the coordination effectiveness of the food safety commissions ultimately depends on a cultural environment that encourages such coordination. Different from D_2_ and D_4_, both D_1_ and D_3_ are not only influenced by the other dimensions but also influence them.

### 5.2. Interrelationships between Different Factors

Table 5 indicates that the level of clarity surrounding the government agencies’ responsibilities as defined by laws and regulations (C_1_), the operability of laws and regulations (C_2_), the authority degree of the food safety commission (C_4_), the clarity degree of the coordination goal (C_5_), and the coordination ability of the commission head (C_6_) have *r_i_* − *c_i_* values greater than zero, so they are causal factors. The amount of attention paid to common goals by the different regulatory government agencies (C_3_), the degree of difference in the positions among the government agencies (C_7_), the cognitive level regarding specialized resource dependency (C_8_), the degree of worry about power erosion (C_9_), the loss degree of a trust culture that encourages coordination (C_10_), the loss degree of a collaborative environment (C_11_), and the extent of concern from social sentiment (C_12_) have *r_i_* − *c_i_* values less than zero, so they are result factors. Moreover, C_6_ is the causal factor with the largest *r_i_* − *c_i_* value. This agrees with the research conclusion drawn in [19]. C_10_ has the smallest *r_i_* − *c_i_* value, indicating that all the other factors in the system have direct or indirect influences on the extent of the loss of a trust culture among the government regulatory agencies. This also indicates that improving the coordination effectiveness of China’s food safety commissions ultimately depends on whether a cultural environment of mutual trust can be established among the different government agencies. This agrees with the research conclusion drawn by Thomson and Perry [26].

Figure 1 describes the interrelationships of the 12 influential factors constituting four dimensions. With dimension D_4_ as an example, the three factors have the following influence relationships: C_12_→C_11_→C_10_. This conforms to the inherent logic that government agencies can reduce transaction costs through collaboration: first, a trial collaboration is conducted; second, a mutual-trust relationship through collaboration is established; and finally, a stable collaboration relationship is established [50]. Because food safety is an issue of relatively high social concern, the transaction costs of interagency collaboration can only be reduced if trust is established gradually through trial collaborations, thereby promoting the establishment of more stable interagency collaboration relationships. This verifies that it is correct to define the coordinating body’s main function as reducing the transaction costs of interagency collaboration. Similarly, the influence relationships among the factors in the other three dimensions can also be expressed and analyzed using the same method as that used for the factors in dimension D_4_.

### 5.3. Key Dimensions Influencing Coordination Effectiveness

Further, the key dimensions influencing the coordination effectiveness can be identified based on the hybrid-weight values obtained using the DANP method. As demonstrated in Table 9, the capability characteristic of the coordinating body (D_2_) has a hybrid weight value of 0.27857, which ranks first among the four dimensions; therefore, D_2_ can be determined as a key dimension. This conclusion prominently shows the importance of the coordinating body’s capability characteristic to improvements in the coordination effectiveness. This agrees with the research conclusion obtained by Ma [18] for the coordination effectiveness of China’s food safety commissions. In addition, the supporting capability of the legal system in guaranteeing coordination (D_1_) has a hybrid-weight value of 0.26528, which ranks second and is relatively close to the influence weight of D_2_, making D_1_ another key dimension. This saliently shows the importance of the legal system and accords with the research conclusion obtained by Zhang et al. [8]. The hybrid-weight values of D_3_ and D_4_ are 0.23521 and 0.22094, respectively, both of which are less than 0.25, taking the third and fourth places, respectively. Thus, D_3_ and D_4_ can be classified as subkey dimensions.

The research results obtained using the DEMATEL method support the above conclusions. The identification of key factors by all scholars using the DEMATEL method is based on the *r_i_* + *c_i_* value [51]. In this paper, D_2_ and D_1_ have *r_i_* − *c_i_* values of 0.16481 and 0.10183, respectively, and *r_i_* + *c_i_* values of 4.47703 and 4.28801, respectively, which are relatively large. This indicates that D_2_ and D_1_ not only have relatively high influences on the other dimensions in the system, but they also have important positions in the system, so they can be regarded as key dimensions. D_3_ and D_4_ have *r_i_* − *c_i_* values of less than zero and relatively small *r_i_* + *c_i_* values, so they can be regarded as subkey dimensions. Therefore, the research conclusions obtained using the DANP method are consistent with those obtained using the DEMATEL method.

### 5.4. Key Factors Influencing Coordination Effectiveness

Table 9 indicates that the coordination ability of the coordinating body head (C_6_), the authority degree (C_4_), the clarity degree of the legal responsibility of the regulatory government agency (C_1_), and the clarity degree of the coordination goal of the coordinating body (C_5_) have hybrid weight values of 0.09448, 0.09388, 0.09245, and 0.09021, respectively, taking the first, second, third, and fourth places, respectively, among the 12 factors, so they can be regarded as the four key factors influencing the coordination effectiveness. The above conclusion agrees with the research conclusions drawn by Eriksson et al. [19], Yasuda [9], Tai [6], Yee and Liu [32], Lindsay et al. [39], and Scott and Merton [29].

The operability of the legal system in terms of guaranteeing coordination (C_2_), the degree of attention to common goals among government agencies (C_3_), and the degree of difference in the positions among the government agencies (C_7_) have hybrid weight values ranging from 0.8 to 0.9. This ranks them at the intermediate level, so they can be regarded as subkey factors. Moreover, the cognitive level of all government agencies regarding specialized resource dependency (C_8_), the degree of a government agency’s worry about power erosion (C_9_), the loss degree of trust culture (C_10_), the loss degree of a collaborative environment (C_11_), and the concern degree for social sentiment (C_12_) have hybrid weight values less than 0.8, so they can be regarded as non-key factors. Note that C_8_, C_9_, C_10_, C_11_, and C_12_ mainly reflect the government agencies’ internal cognition about collaboration and the external influence of the cultural environment. These five factors are more closely related to networked coordination, and they are non-key factors. However, the factors closely related to hierarchical coordination, such as the authority degree of the coordinating body, are key factors. This reflects that each of China’s food safety commissions is a hybrid coordinating body that integrates hierarchical and networked coordination but is dominated by hierarchical coordination. This agrees with the conclusion obtained by Lægreid et al. [14].

With respect to the identified key dimensions, the research conclusion obtained using the DEMATEL method is consistent with that obtained using the DANP method. To further verify the suitability of the DANP method, we determined whether the key factors obtained using these two methods are mutually consistent. If *r_i_* + *c_i_* > 13 is used as the criterion, then C_1_, C_3_, C_4_, C_5_, and C_6_ are the key factors. However, C_3_ has a *r_i_* − *c_i_* value of −0.16567, which is less than zero, so C_3_ is a result factor and should not be determined as a key factor. Therefore, C_1_, C_4_, C_5_, and C_6_ can be determined as key factors based on the DEMATEL method, which is consistent with the conclusion obtained using the DANP method.

This study not only verifies the applicability of DANP, but it also identifies the factors that affect the effectiveness of coordination agencies, providing a theoretical basis for countries around the world to improve the coordination effectiveness of their coordination agencies. At the same time, the research results also reveal some universal patterns: the establishment of mutual trust through cooperation is the only way to establish a stable cooperative relationship, and the coordination mechanism is a hybrid coordination that integrates hierarchical and network coordination, with hierarchical coordination as the main approach. This expands our understanding of the operational mechanism of food safety coordination agencies.

## 6. Conclusions

This paper presents a case study of China’s food safety commissions, and the research findings are as follows:

1. The capability characteristic of the coordinating body and the supporting capability of the legal system to guarantee coordination are key dimensions influencing the coordination effectiveness;
2. The clarity degree of the regulatory government agency’s responsibility as defined by laws and regulations, the authority degree of the food safety commission, the clarity degree of the coordination goal, and the coordination ability of the head are key factors;
3. The four dimensions and 12 factors that influence the coordination effectiveness of China’s food safety commissions are intertwined, constituting a complex network system. In this complex system, all 11 factors directly or indirectly influence the trust culture to different extents, and this verifies that transaction costs are the basis ultimately influencing interagency collaboration.

The above conclusions are supported not only by research conclusions drawn by Chinese scholars but also by findings from the global literature, indicating that the key factors influencing the coordination effectiveness of China’s food safety commissions are similar to the key factors in Western countries, without evident differences due to national situations, social systems, and cultures. The reason is that the purpose of establishing food safety coordinating bodies in China was the same as in Western countries, namely, to solve the problem of the ‘fragmentation’ of governance by multiple government agencies, thereby enhancing the integrity and coordination of public agencies, an approach rooted in the theory and practice of the post-NPM campaign.

The systems of the public management bodies in different countries are different owing to differences in political systems, administrative environments, and cultures [15]. Coordination in centralized countries is different from coordination in countries with pluralist structures [52]. Accordingly, the key factors influencing the coordination effectiveness of China’s food safety commissions are not completely consistent with those influencing the coordination effectiveness in Western countries. The most evident difference is as follows: the research results of this paper indicate that the interagency trust culture and collaborative environment are two non-key factors in China; however, they are important explanatory variables for the coordination effectiveness in Western countries [20]. The cause of the difference may be that the administrative habits are different between China and Western countries. In China, the top–down hierarchical coordination mode has existed for a long time. A study on China by Zhang et al. [8] showed that interagency collaboration primarily relies on formal legal rules, and that informal rules are basically ineffective. In Western countries, however, informal rules, including voluntary initiatives, have also been demonstrated in practice to be important bases for promoting cross-boundary collaborative governance, to which tacit consent has been given by all relevant government agencies [53]. A trust culture and collaborative environment also play important roles in interagency collaboration in Western countries.

The conclusions are helpful for providing a pathway for China to further deepen the reform of its food safety commissions: First, professional persons with food safety knowledge must be chosen to serve as the principal persons-in-charge of the food safety commissions, and these principal persons-in-charge must be kept stable and continuous to some extent. This marks a change from the current practices in most regions, where the principal persons-in-charge are non-professional and transferred frequently. Second, the authority of the offices of China’s food safety commissions should be improved. Recovery of the past practice is suggested; in other words, the food safety commissions should be made into a government organization with independent legal persons and their specialized capabilities should be improved. Third, the supporting capability of the legal system guaranteeing coordination should be enhanced, and the problems of legislation fragmentation, contradictory and split contents between laws, and overlapping, vague, and even blanking responsibilities should be solved in order to form a complete, inter-connecting, and operable system of laws and regulations. Fourth, based on the development requirement of the Chinese path to modernization, and with the aim of achieving a fundamental improvement in the food safety situation, the coordination goals, responsibility scopes, and coordination procedures for the food safety commissions should be clearly defined. This will allow them to focus not only on solving the current problems of high concern from social sentiment, but also to establish a modernized governance system.

This article is one of the few studies on food safety coordination agencies and their influencing factors. Taking China as a case study, the main contributions of this article are as follows: using the DANP method to systematically analyze the interrelationships between the factors that affect the coordination effectiveness of the food safety coordination agencies, revealing the internal mechanisms that affect the coordination effectiveness, and identifying the main influencing factors, providing useful suggestions for improving the coordination effectiveness of the China Food Safety Committee. Moreover, this article provides useful references for other countries to enhance the effectiveness of their food safety coordination agencies and alleviate fragmentation.

This paper also has some limitations. For example, it identifies 12 factors influencing the coordination effectiveness of the food safety coordinating body through a literature study, but there may be other important factors that have been omitted. This is to be improved further in the future. In addition, the DANP method also has some limitations. Although the average deviation rate in measuring the expert consistency was less than 5%, thereby satisfying the requirement of the study using the DANP method, it is debatable whether the expert group represents all stakeholders. In addition, there is an insufficient comparison of the characteristics of the food safety coordination agencies in China and those of other countries, as well as of the factors that affect the coordination effectiveness. Despite this, this study provides a perspective for the academic circle from which to understand the main problems faced in the interagency collaborative governance of food safety risks in China.

## Figures and Tables

**Table 1:** Set of dimensions and factors that influence coordination effectiveness of food safety coordinating body.

Dimensions	Factors
Supporting capability of legal system guaranteeing coordination (D_1_)	Clarity degree of agency’s legal responsibility (C_1_)
	Operability of laws and regulations (C_2_)
	Degree of attention to common goals (C_3_)
Capability characteristic of coordinating body (D_2_)	Authority degree of coordinating body (C_4_)
	Clarity degree of coordination goal (C_5_)
	Coordination ability of coordinating body head (C_6_)
Individual characteristics of government agency in coordination system (D_3_)	Degree of difference in positions among government agencies (C_7_)
	Cognitive level of government agencies on resource dependency (C_8_)
	Degree of government agency’s concern with declining power (C_9_)
Cultural environment encouraging coordination (D_4_)	Loss degree of trust culture (C_10_)
	Loss degree of collaborative environment (C_11_)
	Concern degree from social sentiment (C_12_)

**Table 2:** The direct-relation average matrix (*A*).

	C1	C2	C3	C4	C5	C6	C7	C8	C9	C10	C11	C12
C1	0	3.33333	3.22222	2.88889	3.33333	2.55556	2.88889	2.22222	2.55556	2.33333	2.55556	2.11111
C2	3.55556	0	2.77778	2.88889	3	2.22222	2	2.11111	1.88889	2.22222	2.11111	2.22222
C3	2.66667	2.66667	0	3	2.88889	2.33333	2.33333	2.22222	1.88889	2.55556	2.66667	2.55556
C4	2.33333	3	3.22222	0	3.11111	3.55556	3	2.55556	2.55556	2.44444	2.77778	1.66667
C5	3.11111	2.77778	2.66667	3.11111	0	2.88889	2.66667	2.66667	2.11111	2.22222	2.44444	2.22222
C6	2.77778	2.88889	2.77778	3.77778	3.22222	0	3.55556	2.77778	2.44444	2.22222	2.33333	2
C7	2.22222	2.22222	2.55556	2.77778	2.44444	2.44444	0	2.44444	2.66667	1.88889	2.22222	1.33333
C8	1.66667	1.66667	2.33333	1.88889	2.22222	2.22222	2.44444	0	2.77778	2.33333	2.44444	2
C9	1.88889	1.77778	2.33333	2.33333	2	1.88889	2.33333	2.11111	0	3	2.88889	1.88889
C10	1.66667	2	1.77778	1.77778	1.55556	2	1.77778	2.11111	2.66667	0	3.11111	1.33333
C11	1.88889	1.88889	2.66667	2.11111	2.22222	2.33333	2.11111	2.11111	2.33333	3.11111	0	1.33333
C12	2.22222	2.11111	2.22222	2.44444	2.11111	1.88889	1.77778	1.77778	1.77778	1.88889	1.88889	0

**Table 3:** The normalized initial direct-influence matrix (*D*).

	C_1_	C_2_	C_3_	C_4_	C_5_	C_6_	C_7_	C_8_	C_9_	C_10_	C_11_	C_12_
C_1_	0.00000	0.10830	0.10469	0.09386	0.10830	0.08303	0.09386	0.07220	0.08303	0.07581	0.08303	0.06859
C_2_	0.11552	0.00000	0.09025	0.09386	0.09747	0.07220	0.06498	0.06859	0.06137	0.07220	0.06859	0.07220
C_3_	0.08664	0.08664	0.00000	0.09747	0.09386	0.07581	0.07581	0.07220	0.06137	0.08303	0.08664	0.08303
C_4_	0.07581	0.09747	0.10469	0.00000	0.10108	0.11552	0.09747	0.08303	0.08303	0.07942	0.09025	0.05415
C_5_	0.10108	0.09025	0.08664	0.10108	0.00000	0.09386	0.08664	0.08664	0.06859	0.07220	0.07942	0.07220
C_6_	0.09025	0.09386	0.09025	0.12274	0.10469	0.00000	0.11552	0.09025	0.07942	0.07220	0.07581	0.06498
C_7_	0.07220	0.07220	0.08303	0.09025	0.07942	0.07942	0.00000	0.07942	0.08664	0.06137	0.07220	0.04332
C_8_	0.05415	0.05415	0.07581	0.06137	0.07220	0.07220	0.07942	0.00000	0.09025	0.07581	0.07942	0.06498
C_9_	0.06137	0.05776	0.07581	0.07581	0.06498	0.06137	0.07581	0.06859	0.00000	0.09747	0.09386	0.06137
C_10_	0.05415	0.06498	0.05776	0.05776	0.05054	0.06498	0.05776	0.06859	0.08664	0.00000	0.10108	0.04332
C_11_	0.06137	0.06137	0.08664	0.06859	0.07220	0.07581	0.06859	0.06859	0.07581	0.10108	0.00000	0.04332
C_12_	0.07220	0.06859	0.07220	0.07942	0.06859	0.06137	0.05776	0.05776	0.05776	0.06137	0.06137	0.00000

**Table 4:** The total-influence matrix (*T*).

	C_1_	C_2_	C_3_	C_4_	C_5_	C_6_	C_7_	C_8_	C_9_	C_10_	C_11_	C_12_
C_1_	0.50000	0.60418	0.63949	0.63802	0.63556	0.58364	0.60112	0.55065	0.56758	0.57237	0.60054	0.46544
C_2_	0.55977	0.46217	0.57943	0.58894	0.57934	0.52977	0.53116	0.50448	0.50558	0.52445	0.54178	0.43324
C_3_	0.54266	0.54904	0.50489	0.60038	0.58401	0.54089	0.54822	0.51544	0.51376	0.54215	0.56575	0.44841
C_4_	0.57405	0.59871	0.64369	0.55702	0.63376	0.61528	0.60928	0.56437	0.57215	0.57974	0.61111	0.45588
C_5_	0.57478	0.57247	0.60663	0.62585	0.52021	0.57648	0.57884	0.54726	0.53981	0.55258	0.58019	0.45508
C_6_	0.59524	0.60543	0.64208	0.67695	0.64700	0.52140	0.63420	0.57944	0.57850	0.58220	0.60835	0.47230
C_7_	0.49210	0.49799	0.54057	0.55282	0.53127	0.50571	0.43980	0.48567	0.49897	0.48628	0.51435	0.38366
C_8_	0.44989	0.45519	0.50533	0.49895	0.49587	0.47232	0.48584	0.38689	0.47701	0.47325	0.49384	0.38154
C_9_	0.46186	0.46469	0.51206	0.51734	0.49610	0.46955	0.48856	0.45685	0.40032	0.49836	0.51314	0.38271
C_10_	0.41481	0.42890	0.45209	0.45665	0.43979	0.43059	0.43092	0.41712	0.43976	0.36864	0.47619	0.33410
C_11_	0.46015	0.46590	0.51872	0.50928	0.50023	0.47958	0.48048	0.45490	0.46837	0.49887	0.42481	0.36559
C_12_	0.44222	0.44435	0.47628	0.48786	0.46779	0.43879	0.44182	0.41757	0.42372	0.43494	0.45158	0.30135

**Table 5:** Results of DEMATEL.

Dimensions	*r_i_*	*c_i_*	*r_i_* − *c_i_*	*r_i_* + *c_i_*	Factors	*r_i_*	*c_i_*	*r_i_* − *c_i_*	*r_i_* + *c_i_*
D_1_	2.19492	2.09309	0.10183	4.28801	C_1_	6.95860	6.06753	0.89107	13.02613
					C_2_	6.34012	6.14899	0.19113	12.48911
					C_3_	6.45559	6.62126	−0.16567	13.07685
D_2_	2.32092	2.15611	0.16481	4.47703	C_4_	7.01504	6.71006	0.30498	13.72510
					C_5_	6.73017	6.53093	0.19924	13.26110
					C_6_	7.14308	6.16400	0.97908	13.30708
D_3_	1.90741	2.01516	−0.10775	3.92257	C_7_	5.92919	6.27024	−0.34105	12.19943
					C_8_	5.57592	5.88063	−0.30471	11.45655
					C_9_	5.66155	5.98553	−0.32398	11.64708
D_4_	1.77163	1.93053	−0.15890	3.70216	C_10_	5.08956	6.11385	−1.02429	11.20341
					C_11_	5.62687	6.38162	−0.75475	12.00849
					C_12_	5.22826	4.8793	0.34896	10.10756

**Table 6:** The unweighted supermatrix ( W ).

	C_1_	C_2_	C_3_	C_4_	C_5_	C_6_	C_7_	C_8_	C_9_	C_10_	C_11_	C_12_
C_1_	0.28675	0.34956	0.33989	0.31603	0.32772	0.32302	0.32150	0.31898	0.32105	0.32012	0.31849	0.32448
C_2_	0.34650	0.28861	0.34388	0.32960	0.32640	0.32855	0.32534	0.32274	0.32301	0.33099	0.32247	0.32604
C_3_	0.36675	0.36183	0.31623	0.35437	0.34588	0.34844	0.35316	0.35829	0.35594	0.34889	0.35903	0.34947
C_4_	0.34353	0.34683	0.34799	0.30842	0.36333	0.36684	0.34773	0.34008	0.34885	0.34411	0.34201	0.34986
C_5_	0.34221	0.34118	0.33850	0.35091	0.30200	0.35061	0.33417	0.33798	0.33453	0.33141	0.33593	0.33547
C_6_	0.31425	0.31199	0.31351	0.34068	0.33467	0.28255	0.31810	0.32193	0.31662	0.32448	0.32206	0.31467
C_7_	0.34962	0.34464	0.34754	0.34900	0.34746	0.35388	0.30875	0.35995	0.36304	0.33462	0.34228	0.34434
C_8_	0.32027	0.32733	0.32676	0.32327	0.32851	0.32332	0.34096	0.28664	0.33948	0.32390	0.32406	0.32544
C_9_	0.33011	0.32804	0.32570	0.32773	0.32403	0.32280	0.35029	0.35341	0.29747	0.34148	0.33366	0.33023
C_10_	0.34936	0.34976	0.34836	0.35206	0.34801	0.35012	0.35128	0.35091	0.35745	0.31269	0.38694	0.36615
C_11_	0.36655	0.36131	0.36352	0.37111	0.36539	0.36585	0.37156	0.36618	0.36805	0.40392	0.32950	0.38016
C_12_	0.28409	0.28893	0.28812	0.27684	0.28660	0.28403	0.27715	0.28291	0.27450	0.28339	0.28356	0.25369

**Table 7:** The weighted standardized supermatrix ( $W^{\alpha }$ ).

	C_1_	C_2_	C_3_	C_4_	C_5_	C_6_	C_7_	C_8_	C_9_	C_10_	C_11_	C_12_
C_1_	0.07172	0.08743	0.08501	0.08453	0.08765	0.08639	0.07880	0.07818	0.07869	0.07597	0.07559	0.07701
C_2_	0.08666	0.07218	0.08601	0.08815	0.08730	0.08787	0.07974	0.07910	0.07917	0.07855	0.07653	0.07738
C_3_	0.09173	0.09050	0.07909	0.09478	0.09251	0.09319	0.08656	0.08781	0.08724	0.08280	0.08521	0.08294
C_4_	0.08911	0.08996	0.09027	0.07933	0.09346	0.09436	0.08674	0.08484	0.08702	0.08049	0.08000	0.08184
C_5_	0.08877	0.08850	0.08780	0.09026	0.07768	0.09019	0.08336	0.08431	0.08345	0.07752	0.07858	0.07847
C_6_	0.08151	0.08093	0.08132	0.08763	0.08609	0.07268	0.07935	0.08031	0.07898	0.07590	0.07534	0.07361
C_7_	0.08920	0.08793	0.08867	0.09235	0.09194	0.09364	0.07422	0.08653	0.08727	0.08027	0.08210	0.08260
C_8_	0.08171	0.08351	0.08337	0.08554	0.08692	0.08555	0.08197	0.06891	0.08161	0.07769	0.07773	0.07806
C_9_	0.08422	0.08369	0.08310	0.08672	0.08574	0.08541	0.08421	0.08496	0.07151	0.08191	0.08004	0.07921
C_10_	0.09001	0.09011	0.08975	0.09289	0.09182	0.09238	0.08772	0.08763	0.08926	0.07155	0.08854	0.08378
C_11_	0.09443	0.09308	0.09365	0.09791	0.09640	0.09653	0.09279	0.09144	0.09191	0.09242	0.07539	0.08698
C_12_	0.07319	0.07444	0.07423	0.07304	0.07562	0.07494	0.06921	0.07065	0.06855	0.06484	0.06488	0.05805

**Table 8:** The stable-limit supermatrix ( $W^{*}$ ).

	C_1_	C_2_	C_3_	C_4_	C_5_	C_6_	C_7_	C_8_	C_9_	C_10_	C_11_	C_12_
C_1_	0.08059	0.08059	0.08059	0.08059	0.08059	0.08059	0.08059	0.08059	0.08059	0.08059	0.08059	0.08059
C_2_	0.08158	0.08158	0.08158	0.08158	0.08158	0.08158	0.08158	0.08158	0.08158	0.08158	0.08158	0.08158
C_3_	0.08783	0.08783	0.08783	0.08783	0.08783	0.08783	0.08783	0.08783	0.08783	0.08783	0.08783	0.08783
C_4_	0.08639	0.08639	0.08639	0.08639	0.08639	0.08639	0.08639	0.08639	0.08639	0.08639	0.08639	0.08639
C_5_	0.08406	0.08406	0.08406	0.08406	0.08406	0.08406	0.08406	0.08406	0.08406	0.08406	0.08406	0.08406
C_6_	0.07955	0.07955	0.07955	0.07955	0.07955	0.07955	0.07955	0.07955	0.07955	0.07955	0.07955	0.07955
C_7_	0.08634	0.08634	0.08634	0.08634	0.08634	0.08634	0.08634	0.08634	0.08634	0.08634	0.08634	0.08634
C_8_	0.08108	0.08108	0.08108	0.08108	0.08108	0.08108	0.08108	0.08108	0.08108	0.08108	0.08108	0.08108
C_9_	0.08259	0.08259	0.08259	0.08259	0.08259	0.08259	0.08259	0.08259	0.08259	0.08259	0.08259	0.08259
C_10_	0.08793	0.08793	0.08793	0.08793	0.08793	0.08793	0.08793	0.08793	0.08793	0.08793	0.08793	0.08793
C_11_	0.09185	0.09185	0.09185	0.09185	0.09185	0.09185	0.09185	0.09185	0.09185	0.09185	0.09185	0.09185
C_12_	0.07022	0.07022	0.07022	0.07022	0.07022	0.07022	0.07022	0.07022	0.07022	0.07022	0.07022	0.07022

**Table 9:** Normalized hybrid weights of dimensions and factors that influence coordination effectiveness of food safety coordinating body.

	Hybrid Weights	Ranks	Criteria	Hybrid Weights	Ranks
D_1_	0.26528	2	C_1_	0.09245	3
			C_2_	0.08533	6
			C_3_	0.08750	5
D_2_	0.27857	1	C_4_	0.09388	2
			C_5_	0.09021	4
			C_6_	0.09448	1
D_3_	0.23521	3	C_7_	0.08120	7
			C_8_	0.07639	10
			C_9_	0.07762	9
D_4_	0.22094	4	C_10_	0.07164	11
			C_11_	0.07842	8
			C_12_	0.07088	12

## Data Availability

The original contributions presented in the study are included in the article, further inquiries can be directed to the corresponding author.
